# Genome Sequence of the Acidophilic Ferrous Iron-Oxidizing Isolate *Acidithrix ferrooxidans* Strain Py-F3, the Proposed Type Strain of the Novel Actinobacterial Genus *Acidithrix*

**DOI:** 10.1128/genomeA.00382-15

**Published:** 2015-04-30

**Authors:** Sebastian Eisen, Anja Poehlein, D. Barrie Johnson, Rolf Daniel, Michael Schlömann, Martin Mühling

**Affiliations:** a Institute of Biological Sciences, TU Bergakademie Freiberg, Freiberg, Germany; b Genomic and Applied Microbiology & Göttingen Genomics Laboratory, Georg-August-Universität Göttingen, Göttingen, Germany; c College of Natural Sciences, Bangor University, Bangor, United Kingdom

## Abstract

Extremely acidophilic iron-oxidizing Gram-positive bacteria comprise species within the phyla *Firmicutes* and *Actinobacteria*. Here, we report the 4.02-Mb draft genome of *Acidithrix ferrooxidans* Py-F3, which was isolated from a stream draining an abandoned copper mine and proposed as the type species of a new genus of *Actinobacteria*.

## GENOME ANNOUNCEMENT

Strain Py-F3 is a filamentous Gram-positive acidophilic bacterium that was isolated from an acidic (pH 2.5) metal-rich stream draining an abandoned copper mine in north Wales (1). Based on a comparison of its 16S rRNA gene sequence, the isolate was identified as a member of the *Actinobacteria*. However, the low 16S rRNA gene sequence similarity (91 to 93%) to known species indicates that the isolate belongs to a new genus designated *Acidithrix*, with isolate Py-F3 proposed as the type strain of the novel species *Acidithrix ferrooxidans* (2). Like all other known extremely acidophilic actinobacteria*, A. ferrooxidans* can reduce ferric iron, and, like most species, it can also oxidize ferrous iron (2).

The MasterPure complete DNA purification kit (Epicentre) was used to isolate the chromosomal DNA of *A. ferrooxidans* strain Py-F3. Genome sequencing was carried out employing a hybrid approach using the 454 GS-FLX system (Titanium GS70 chemistry; Roche Life Science) and the Genome Analyzer II (Illumina). Overall, 137,313 454-shotgun and 7,304,600 Illumina paired-end reads were produced, amounting to 18.4-fold and 203-fold estimated genome coverages, respectively. The assembly was performed with the Roche Newbler assembly software 2.9 and MIRA (3) and resulted in a total of 183 contigs. The draft genome sequence of strain Py-F3 indicates a genome size of 4.02 Mb and an overall G+C content of 47.8 mol%. The Integrated Microbial Genomes Expert Review (IMG-ER) system (4) was used for the initial annotation of the 3,703 predicted protein-coding genes and manually curated using the Swiss-Prot, TrEMBL, and InterPro databases (5). The genome harbors 8 rRNA genes and 46 tRNA genes, which were identified with RNAmmer (6) and tRNAscan (7), respectively.

Analyses of the protein-coding genes revealed that *A. ferrooxidans* strain Py-F3 appears to utilize sulfate as a sole source of sulfur and amino acids as a nitrogen source. The finding regarding its use of amino acids is supported by the inability of the strain to grow in media without complex constituents, such as yeast extract (2). Moreover, *A. ferrooxidans* strain Py-F3 contains a urease gene cluster that might play a role in pH homeostasis, as described for *Helicobacter pylori* (8). This hypothesis is based on the fact that urea hydrolysis products (ammonium and bicarbonate) provide a means for buffering intracellular protons in order to survive, e.g., under short-term instances of particularly acidic conditions (9). If pH homeostasis is indeed the role of the urease in *A. ferrooxidans* strain Py-F3, then this would, however, entail intracellular production and storage of urea, since no corresponding ABC transporter for the uptake of urea from the environment was detected in the genome.

The genome sequence of *A. ferrooxidans* strain Py-F3 encodes the two subunits of a type I RubisCO and several enzymes required for carbon fixation via the Calvin-Benson-Bassham cycle. Whether *A. ferrooxidans* strain Py-F3 is indeed capable of fixing CO_2_, however, has not yet been tested.

### Nucleotide sequence accession numbers. 

The results from this genome sequencing project have been deposited at GenBank under the accession no. JXYS00000000. The version described in this paper is version JXYS01000000.
